# Local Resonant Attenuation of Stress Waves in Particulate Composites

**DOI:** 10.3390/ma14112991

**Published:** 2021-06-01

**Authors:** Dandan Xu, Yu Guo

**Affiliations:** 1 College of Mechanical Engineering, Zhejiang University of Technology, Hangzhou 310023, China; xudandan@zjut.edu.cn; 2 Department of Engineering Mechanics, Zhejiang University, Hangzhou 310027, China

**Keywords:** local resonance, stress wave, attenuation, particulate composite

## Abstract

The attenuation of stress waves due to the local resonance is numerically studied using the finite element method (FEM) in this work. The natural frequency of a representative composite unit embedded with coated particles is analyzed and the major factors that influence the natural frequency are examined. Local resonance is inspired when the frequency of the incident stress wave is close to the natural frequency of the particles in the composite. Significant reduction in the amplitude of the stress is obtained when the local resonance occurs, because a large amount of the incident energy is converted to the kinetic energy of the particles, which is rapidly dissipated through the strong oscillations of those particles. It is also observed that the attenuation for the incident stress waves with a range of frequencies can be achieved by using the particles with various local natural frequencies in a composite.

## 1. Introduction

Energy localization is an effective approach to attenuate stress waves. By designing the microstructures in a material, a large amount of energy of the incident stress waves can be locally trapped in some specific parts of the material, and the energy stream that propagates forward is reduced, leading to a reduction in the amplitude of the stress wave. Local deformation, motion, or failure of the material may cause energy localization [1]. Achieving the stress wave attenuation by promoting the local resonance of the microstructures of the composite is of great interests for the development of high-performance protection composite materials.

Concept of local resonance is widely used in the research on photonic crystals [2,3]. Liu et al. [4] fabricated sonic crystals, based on the idea of localized resonant, the structure cell consists of the high-density solid core and elastic soft coating. The material preforms negative modulus at a certain frequency range. Such materials with negative elastic modulus belong to metamaterials [5], which have physical properties that the natural materials do not possess due to special artificial design. It is found that the sonic crystals with local resonance structure have strong attenuation band gaps to the specific frequency of sound wave [6]. Thus, the acoustic waves of some particular frequencies are unable to propagate through the sonic crystals. Dupont et al. [7] presented a cubic array of thick spherical shells with holes in air, and observed a low frequency stop band of the acoustic wave.

The phenomenon of band gap and the design of metamaterials also exist in mechanical materials [8,9]. Li and Wang [10] proposed a two-dimensional mechanical system containing one rigid body and several springs around it as an elastic metamaterial exhibiting both negative mass and negative modulus under specific frequencies. The local resonance mechanism is also used for elastic wave absorption [11], vibration suppression [12], and cloaking [13]. Mitchell et al. [14] proposed a type of concrete called metaconcrete for the attenuation of elastic waves induced by dynamic excitation. Kettenbeil and Ravichandran [15] carried out experimental investigation of the dynamic behavior of metaconcrete, and found that the locally resonant metamaterial can attenuate the applied stress waves at the inclusion’s eigenfrequencies. Nouh et al. [16] presented metamaterial plates with built-in local resonances consisting of a viscoelastic membrane and a small mass, and the plates are effective in attenuating and filtering low-frequency structural vibration.

The present work is performed based on the previous work for a deeper understanding of the mechanism of the stress wave attenuation by the addition of solid particles in a composite: a two-dimensional composite model with coated particles is setup in the finite element method (FEM) simulations and the effects of the properties of particles and coatings on the attenuation of stress wave are explored. The frequency of the stress wave in this paper is in the order of MHz, which is much larger than that in the previous studies (e.g., kHz). In addition, a novel study is performed on the composite that can simultaneously attenuate a combination of stress waves of different frequencies.

This paper is organized as follows: the natural frequency of the composite and its influence factors are discussed at first, and some theoretical and numerical work are introduced in Section 2; the stress wave propagation in the composite is simulated, and the stress wave attenuation effect of the material is verified in Section 3; the design of multi-frequency attenuation material is discussed in Section 4. The conclusions are drawn in Section 5.

## 2. Natural Frequency of the Particulate Composite

### 2.1. Theoretical Model

**Figure 1:**
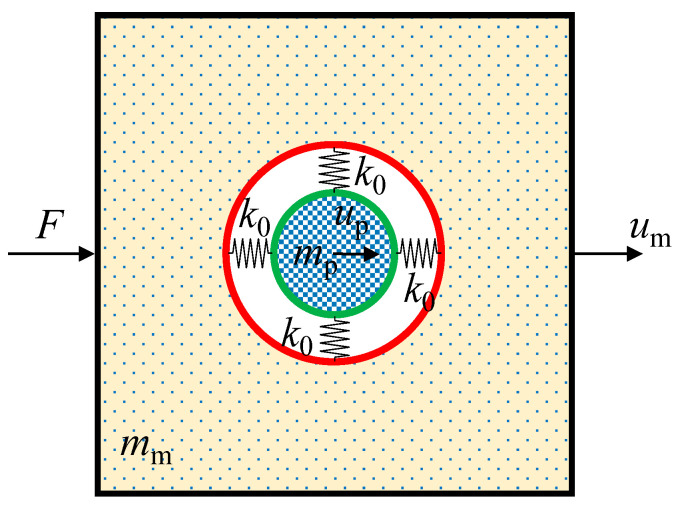
Vibrator model of a coated particle embedded in a matrix.

Figure 1 shows an illustration of a two-dimensional (2D) model of a unit cell embedded with a coated particle. In the theoretical analysis, the matrix and the particle are assumed to be rigid and the annular coating is treated as a deformable material with negligible mass. The elastic support of the coating to the particle is simplified as four uniformly distributed springs with the stiffness of *k*_0_. The stiffness of the coating depends on the Young’s modulus and thickness. The masses of the matrix and the particle are *m_m_* and *m_p_*, respectively. A load of ***F*** is exerted on the left-hand side boundary and the other boundaries are load-free with no constrained displacements. In the process, the displacements in the horizontal direction of the matrix and particle are denoted as *u_m_* and *u_p_*, respectively.

The equations that govern the motion of the matrix and particle are obtained according to Newton’s second law of motion,

(1) $$\left[\begin{array}{cc} m_{m} & 0\\ 0 & m_{p} \end{array}\right]\left(\begin{array}{c} {\ddot{u}}_{m}\\ {\ddot{u}}_{p} \end{array}\right)+\left[\begin{array}{cc} 2k_{0} & -2k_{0}\\ -2k_{0} & 2k_{0} \end{array}\right]\left(\begin{array}{c} u_{m}\\ u_{p} \end{array}\right)=\left(\begin{array}{c} F\\ 0 \end{array}\right)$$

Supposing $F=F_{0}e^{i\omega t}$, $\left(\begin{array}{c} u_{m}\\ u_{p} \end{array}\right)=\left(\begin{array}{c} u_{m0}\\ u_{p0} \end{array}\right)e^{i\omega t}$, then,

(2) $$\left[\begin{array}{cc} 2k_{0}-m_{m}\omega^{2} & -2k_{0}\\ 2k_{0} & -\left(2k_{0}-m_{p}\omega^{2}\right) \end{array}\right]\left(\begin{array}{c} u_{m0}\\ u_{p0} \end{array}\right)=\left(\begin{array}{c} F_{0}\\ 0 \end{array}\right)$$

which gives

(3) $$\frac{u_{m0}}{F_{0}}=\frac{2k_{0}-m_{p}\omega^{2}}{\left(2k_{0}-m_{m}\omega^{2}\right)\left(2k_{0}-m_{p}\omega^{2}\right)-4k_{0}^{2}}$$

(4) $$\frac{u_{p0}}{F_{0}}=\frac{2k_{0}}{\left(2k_{0}-m_{m}\omega^{2}\right)\left(2k_{0}-m_{p}\omega^{2}\right)-4k_{0}^{2}}.$$

The idea for the optimal design of stress wave attenuation is to allow the particles to absorb as much energy as possible, and meanwhile the movement and the kinetic energy of the matrix are minimized.

In Equation (3), if the external frequency of excitation satisfies

(5) $$\omega =\sqrt{\frac{2k_{0}}{m_{p}}}$$

i.e., the frequency of the excitation is equal to the natural frequency of the local particle vibration mode, we have

(6) $$\frac{u_{m0}}{F_{0}}=0,$$

The equivalent mass of the structure is,

(7) $$M_{\mathrm{eff}}=\frac{F}{{\ddot{u}}_{m}}=-\frac{1}{\omega^{2}}\frac{\left(2k_{0}-m_{m}\omega^{2}\right)\left(2k_{0}-m_{p}\omega^{2}\right)-4k_{0}^{2}}{2k_{0}-m_{p}\omega^{2}}.$$

Thus, the equivalent mass is toward infinity, indicating that the matrix does not move under the effect of load.

It can be seen that the natural frequency of the model is mainly determined by the stiffness of the coating, *k*_0_, and the mass of the particle, *m_p_*. This statement is verified by the following numerical simulations, and the relations between these quantities and natural frequency are further discussed in this work.

### 2.2. Numerical Model

**Figure 2:**
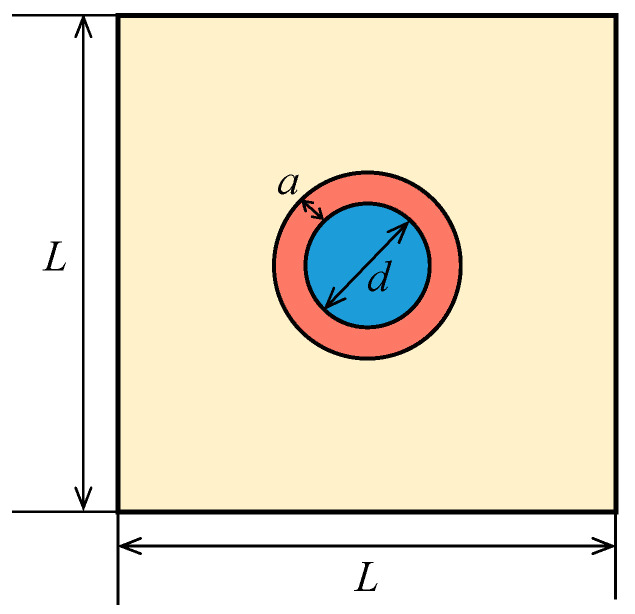
Coated vibrator model in the FEM simulations.

Numerical vibrator model for natural frequency analysis is created and illustrated in Figure 2, where *L* is the length of the square model, *d* is the diameter of the particle, and *a* is the thickness of the coating.

The FEM commercial software ABAQUS (Version 6.14) is used to perform the simulations. In the numerical model, *L* = 200 μm, *d* = 50 μm, *a* = 10 μm. The matrix material is based on resemble polyurea, which is a polymer with viscosity. In this work, the matrix is considered as an elastic material for simplicity and its Young’s modulus is assigned as the storage modulus of the resemble polyurea. The particles are glass beads. The coating parameters are based on those of silastic, while the Young’s modulus of the coating is adjusted to achieve various natural frequencies. The base material parameters used in the simulations are shown in Table 1.

**Figure 3:**
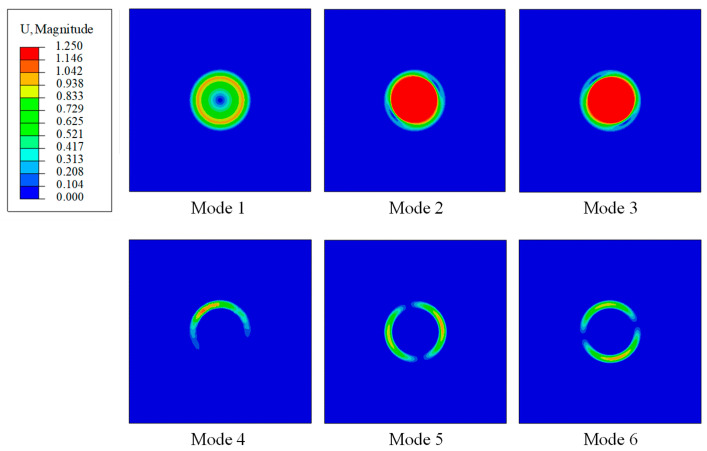
First six orders of natural modes of the coated vibrator model (mm).

Figure 3 shows the first six orders of the natural modes obtained by the frequency analyses. All these six modes are local displacement modes. The first three modes represent the motions of the particle: Mode 1 is the rotation of the particle, Modes 2 and 3 represent the translational motion of the particle in two perpendicular directions. It is found that the frequencies corresponding to Mode 2 and 3 are very similar. Modes 4 to 6 represent the deformation of the coating. The translational mode of the particle is related to the vibration of the particle, which is used to achieve the energy localization. Therefore, the translational mode of the particle is of the great interest, and in the following, the natural frequency is referred to as that of the Mode 2.

### 2.3. Determination of the Natural Frequency

**Figure 4:**
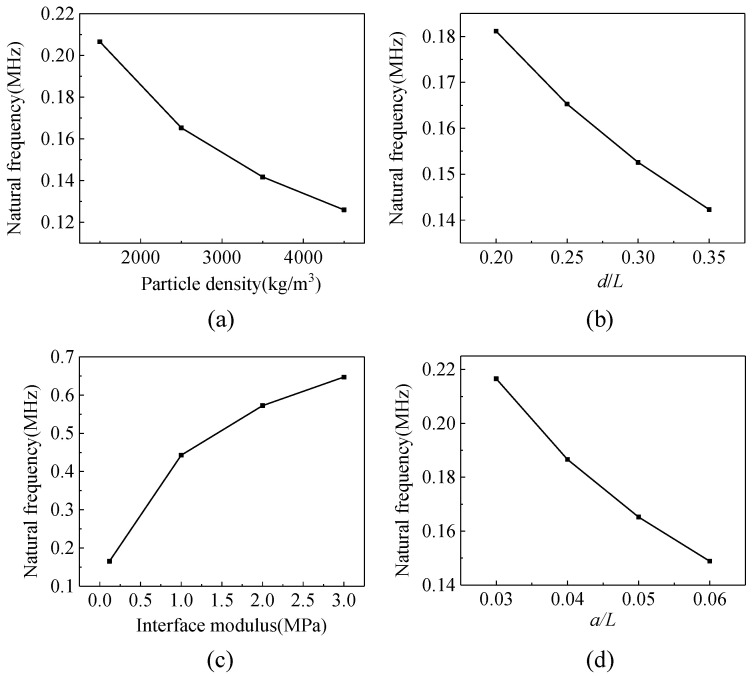
The effects of (**a**) particle density, (**b**) particle diameter, (**c**) interface Young’s modulus of the coating, and (**d**) thickness of the coating on the natural frequency.

In this section, the factors that influence the natural frequency are investigated numerically. According to the theoretical analyses, natural frequency depends on the mass of the particle and the stiffness of the coating, therefore factors that are relevant to them more likely affect the natural frequency. The effects of the particle density, particle diameter, Young’s modulus, and thickness of the coating are considered. The simulation results are shown in Figure 4.

Figure 4a,b shows the influences of the particle density and diameter on the natural frequency, respectively. The natural frequency decreases with the increase in these two parameters, which leads to an increase in the mass of the particle.

The mass of the particle can be controlled by adjusting the density and size, and the stiffness of the system is determined by the properties of the coating. Young’s modulus is an important factor. As shown in Figure 4c, the increase of the modulus of coating can increase the natural frequency. However, an important prerequisite for the model to have local translational modes of the particle is that the modulus of the coating is much smaller than those of the matrix and the particle. Thus, an upper limit exists for the natural frequency as the modulus of the coating is limited.

Another way to adjust the stiffness is to change the thickness of the coating. As shown in Figure 4d, the increase of the coating thickness decreases the natural frequency. The coating can be analogous to the tandem springs in the thickness direction: more springs lead to lower stiffness. Therefore, the stiffness of the coating decreases as the thickness increases.

Based on the understanding of the effects of these factors, the natural frequency of the composite system can be adjusted to match the frequency of the excitation.

## 3. Stress Wave Attenuation

### 3.1. FEM Model

In this section, FEM models are generated by ABAQUS (Version 6.14, SIMULIA by Dassault Systèmes, Providence, RI, USA) to simulate the stress wave propagation in the composite embedded with multiple coated particles. Based on the simulation results, the stress attenuation in different materials is evaluated and the effectiveness of the use of the local resonance mechanism to attenuate the stress wave is examined.

**Figure 5:**
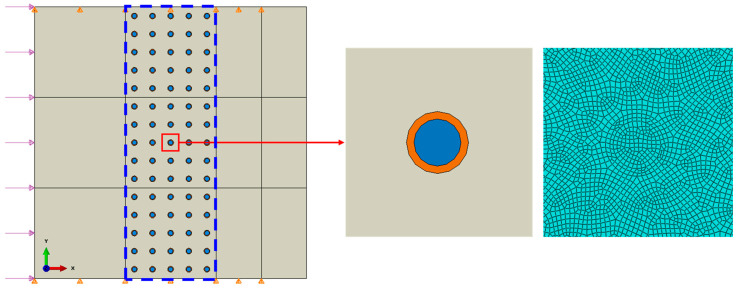
Schematic diagram of the FEM model of the composite embedded with multiple coated particles and the local zoom-in mesh diagram.

The numerical model is shown in Figure 5. The dimensions of the model are 3 mm × 3 mm, in which the coated particles are arranged in the structural grids in the center of the composite. The diameter of each particle is 50 μm, the thickness of the coating is 8 μm, and the distance between the centers of the particles is 200 μm in both horizontal and vertical directions. In the simulation, the Young’s modulus of the coating is 1.18 MPa, and other material parameters are the same to those presented in Table 1. A uniform sinusoidal stress wave with the amplitude, *p*_0_, of 0.4 MPa is exerted on the left-hand side boundary. The displacements in the vertical direction (*y* direction) of the upper and lower boundaries are restrained. The zoom-in diagram on the right in Figure 5 shows the local mesh around the particle region. The plane strain element CPE4R is used except the right-hand boundary, for which infinite element CINPE 4 is used in order to avoid the influence of the reflected wave. Dynamic, explicit analysis scheme was used in the simulations.

### 3.2. Results

**Figure 6:**
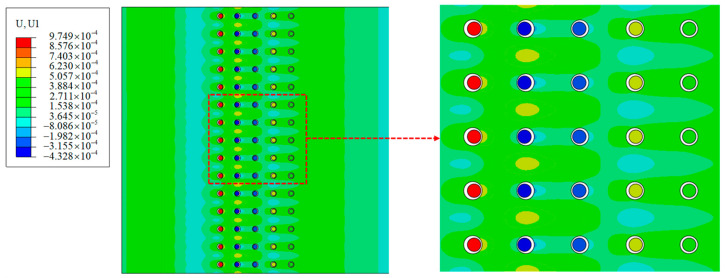
Displacement field in horizontal direction (mm) (*f* = 0.6 MHz, *t* = 0.8 μs, the insert is the zoom-in view of the region wrapped by the box).

Based on the frequency analyses discussed in Section 2.2, the natural frequency of the coated particle cell in this model is calculated as 0.6 MHz. To match the frequency of the incident stress wave with the natural frequency, the stress wave of 0.6 MHz is applied, so that local resonance is expected to be inspired. The propagation process of the stress wave is simulated, and the field of displacements at the time t = 0.8 μs is shown in Figure 6. For a clearer observation of the movement of the particle, the coating is hidden in the figure. As shown in the enlarged view on the right-hand side, the displacement field within the particle is uniform, which means little deformation occurs to the particle. The displacement of the particles is much larger than that of the matrix, and the deformation mainly occurs in the coating. The dynamics of the system is similar to the vibrating process of a mass-spring system, in which the coating plays a role of a flexible support and the particle is the oscillator. The frequency of the incident wave is 0.6 MHz, equal to the natural frequency of the coated particle. As a result, the excitation inspires the local resonance of the particles, and a large percentage of the external work transforms to the kinetic energies of the particles.

**Figure 7:**
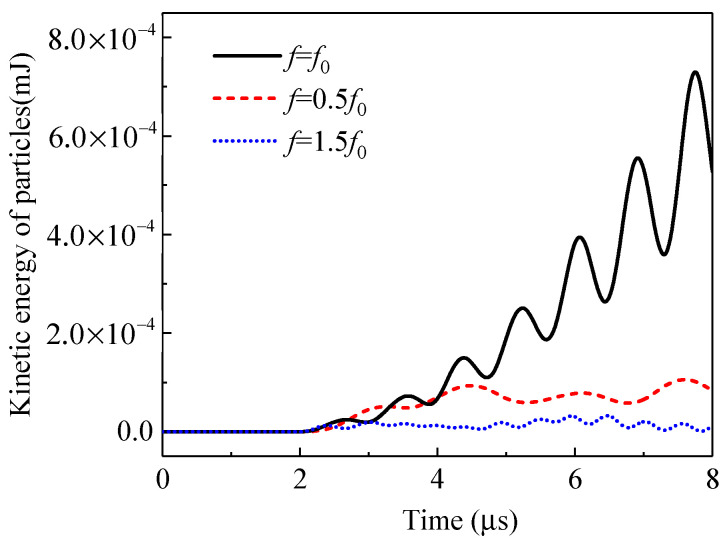
Time evolution of the total kinetic energies of the particles subject to the stress waves of different frequencies.

Besides *f* = 0.6 MHz, the frequencies of *f* = 0.3 MHz and *f* = 0.9 MHz are also considered in the simulations. The total kinetic energies of all the particles for these frequencies are plotted in Figure 7. The maximum kinetic energy is obtained when the frequency of the excitation is equal to that of the system, i.e., *f* = *f*_0_. When resonance is inspired, the vibration frequency of the particles is the same as the excitation and the amplitude of vibration achieves the maximum. As the incident wave from the left-hand side boundary is a sinusoidal wave, the total kinetic energy of the particles evolves with time in a sinusoidal pattern accordingly.

**Figure 8:**
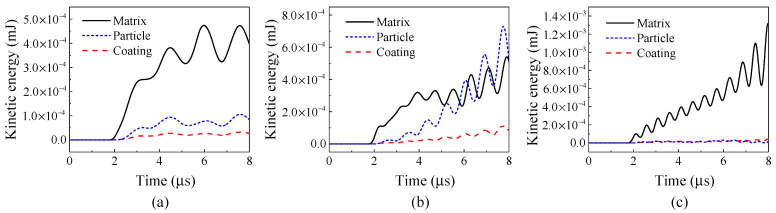
Kinetic energies of matrix, particles and coating with (**a**) *f* = 0.3 MHz, (**b**) *f* = 0.6 MHz, and (**c**) *f* = 0.9 MHz.

For a further analysis, the blue dashed box in Figure 5 is selected as a window of observation. Figure 8 shows the kinetic energies of the matrix, particles, and coating in the window under the stress waves of various frequencies. It is evident that the incident frequency of the stress wave has a significant impact on the partition of the kinetic energies among the matrix, particles, and coating, indicating that the change of the input frequency causes the change of the displacement and deformation modes of the composite system.

When the incident frequency is equal to the natural frequency of the coated particle cell, i.e., *f* = *f*_0_, the kinetic energy of the particles can exceed that of the matrix, while in the other two cases, the kinetic energy of the matrix is much larger than those of the particles and coating. The larger proportion of kinetic energy occupied by the particles indicates that the vibrating motion of particles is significant. These results show that the incident stress wave can excite the local resonance of the particles when the incident frequency is close to the natural frequency of the translational mode of the particle. The present simulation results are consistent with the previous experimental observation by Kettenbeil and Ravichandran [15].

The significant difference is that when the frequency of the incident stress wave is 0.3 MHz or 0.9 MHz, the kinetic energy of particles is much less, and the motion of the particles is very limited.

### 3.3. Stress Wave Attenuation Coefficient

**Figure 9:**
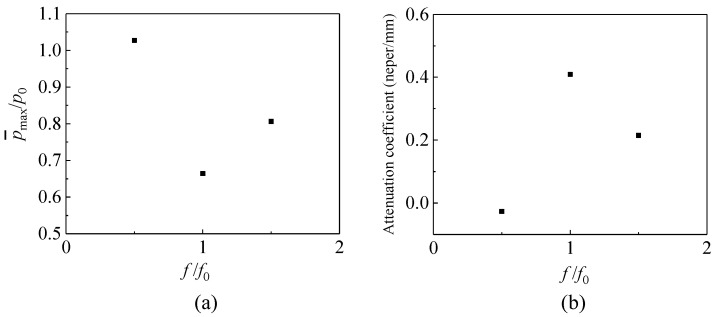
(**a**) Output stress wave value; (**b**) stress attenuation coefficient.

In the above section, stress wave attenuation is analyzed from the point of view of the kinetic energy. In this section, the stress is explored to evaluate the attenuation of the wave. The peak value ${\bar{p}}_{\max}$ of the average normal stress on the right-hand side boundary of the observation window in Figure 5 is measured. The ratio of ${\bar{p}}_{\max}$ to the amplitude of the incident wave *p*_0_ is employed to quantify the attenuation capacity of this structure. Lower ${\bar{p}}_{\max}/p_{0}$ indicates a greater decrease in stress amplitude and a larger attenuation. The simulation results are shown in Figure 9a.

In order to evaluate the attenuation property of the material, an attenuation coefficient is defined as

(8) $$\alpha =\frac{ln\left(p_{0}/{\bar{p}}_{\max}\right)}{\Delta L},$$

where Δ*L* is the distance between the left-hand side and right-hand side boundaries of the dashed region as shown in Figure 5.

When the frequency of the incident wave is 0.6 MHz, i.e., the same as the natural frequency, the peak value of the stress at the output section is much lower than that with *f* = 0.3 MHz and *f* = 0.9 MHz. The attenuation of the stress wave at 0.6 MHz is greater than that with the other two frequencies. It indicates that the material can attenuate the stress wave most with the wave frequency close to the local natural frequency.

To design a composite for the attenuation of stress wave, the natural frequency of the coated particle system can be tuned to match the frequency of the incident stress wave, in order to achieve the best attenuation performance.

## 4. Designed Composite for Multi-Frequencies Attenuation

Composite with uniform microstructures has only one natural frequency of Mode 2 (Figure 3) and it is effective for the attenuation of the incident stress wave with similar frequency. Therefore, the frequency range of the stress waves that can be attenuated is quite limited. How to conquer this disadvantage and achieve the attenuation on a wide range of stress waves is discussed in this section.

**Figure 10:**
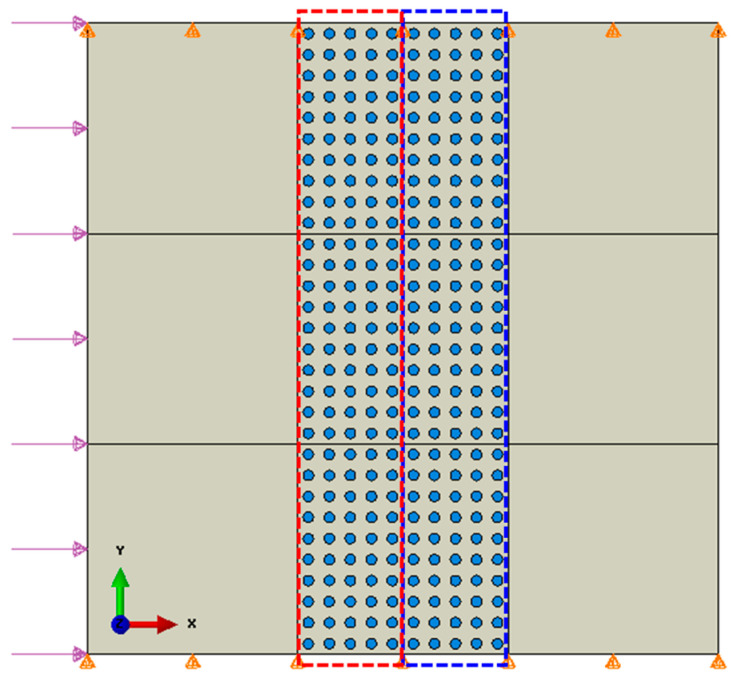
Numerical model of the composite with two different local natural frequencies: $f_{0}\approx 0.3\;\mathrm{MHz}$ for the particles in the red rectangular box and $f_{0}\approx 0.6\;\mathrm{MHz}$ for the particles in the blue rectangular box.

In the numerical model shown in Figure 10, the particles in the red rectangular box have the local natural frequency of $f_{0}\approx 0.3\;\mathrm{MHz}$ and the particles in the blue rectangular box have the local natural frequency of $f_{0}\approx 0.6\;\mathrm{MHz}$. The natural frequencies of the local vibration modes are determined by adjusting the Young’s modulus of the coating: *E* = 0.3 MPa for $f_{0}\approx 0.3\;\mathrm{MHz}$ and *E* = 1.5 MPa for $f_{0}\approx 0.6\;\mathrm{MHz}$. Incident stress waves with the frequencies of 0.3 MHz and 0.6 MHz are applied on the left-hand side boundary of the model.

**Figure 11:**
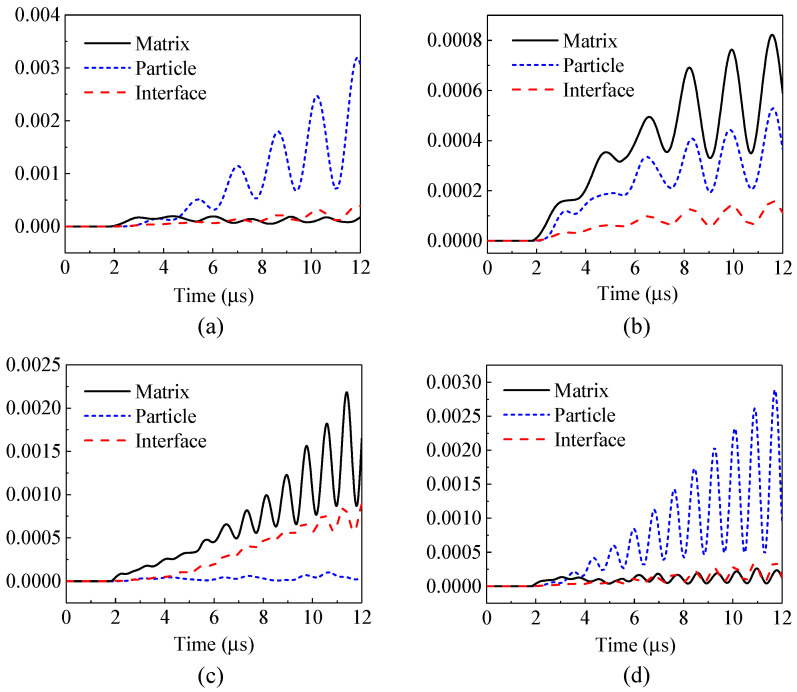
Kinetic energies (mJ): (**a**) *f*_0_ = 0.3 MHz, *f* = 0.3 MHz; (**b**) *f*_0_ = 0.3 MHz, *f* = 0.6 MHz; (**c**) *f*_0_ = 0.6 MHz, *f* = 0.3 MHz; (**d**) *f*_0_ = 0.6 MHz, *f* = 0.6 MHz.

**Figure 12:**
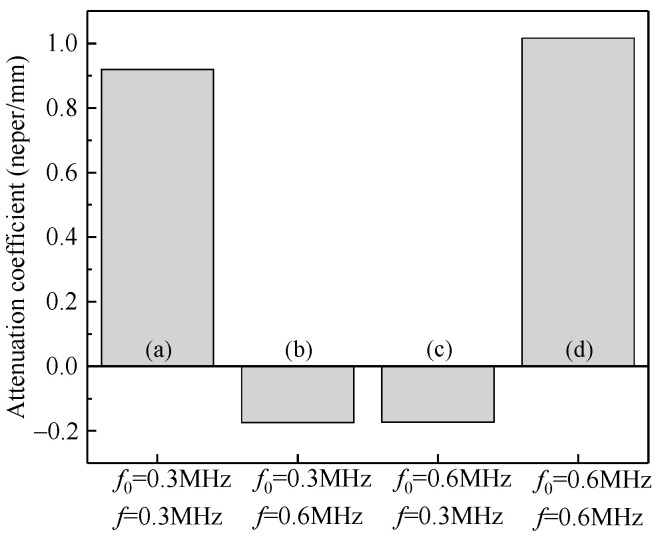
Attenuation coefficient of the composites with a single local natural frequency of particles: (**a**) *f*_0_ = 0.3 MHz, *f* = 0.3 MHz; (**b**) *f*_0_ = 0.3 MHz, *f* = 0.6 MHz; (**c**) *f*_0_ = 0.6 MHz, *f* = 0.3 MHz; (**d**) *f*_0_ = 0.6 MHz, *f* = 0.6 MHz.

First, the kinetic energies and the attenuation coefficients of the composites containing only a single local natural frequency of particles are shown in Figure 11 and Figure 12. Figure 11 illustrates that when the frequency of the incident stress wave is the same as the natural frequency (e.g., Figure 11a: *f*_0_ = 0.3 MHz, *f* = 0.3 MHz, and Figure 11d: *f*_0_ = 0.6 MHz, *f* = 0.6 MHz), the kinetic energies of the vibrating particles are dominant, indicating the occurrence of local resonance and a better performance of attenuation. Figure 12 also confirms that the attenuation coefficients of the composite to the stress wave are larger in these two cases. 

When the frequency of the incident stress wave deviates from the natural frequency of the system, e.g., Figure 11b: *f*_0_ = 0.3 MHz, *f* = 0.6 MHz, and Figure 11c: *f*_0_ = 0.6 MHz, *f* = 0.3 MHz, the energy of the matrix is more dominant, and the composite exhibits no attenuation on the stress waves in these two cases, as shown in Figure 12.

**Figure 13:**
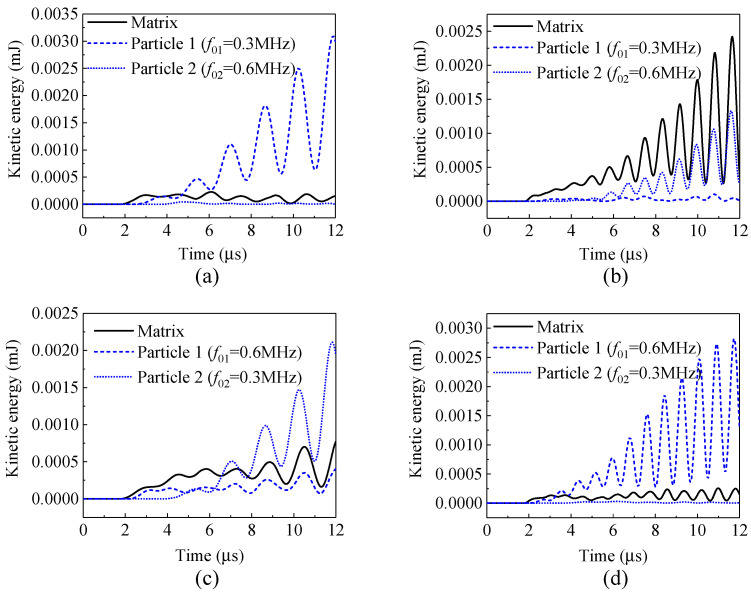
Kinetic energy (mJ): (**a**) *f*_01_ = 0.3 MHz, *f*_02_ = 0.6 MHz, *f* = 0.3 MHz; (**b**) *f*_01_ = 0.3 MHz, *f*_02_ = 0.6 MHz, *f* = 0.6 MHz; (**c**) *f*_01_ = 0.6 MHz, *f*_02_ = 0.3 MHz, *f* = 0.3 MHz; (**d**) *f*_01_ = 0.6 MHz, *f*_02_ = 0.3 MHz, *f* = 0.6 MHz.

**Figure 14:**
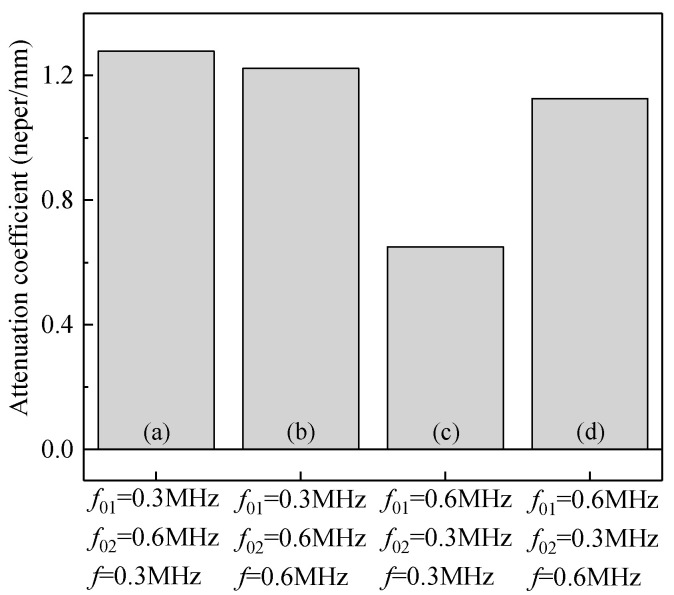
Attenuation coefficients of the composites embedded with two types of particles of different natural frequencies: (**a**) *f*_01_ = 0.3 MHz, *f*_02_ = 0.6 MHz, *f* = 0.3 MHz; (**b**) *f*_01_ = 0.3 MHz, *f*_02_ = 0.6 MHz, *f* = 0.6 MHz; (**c**) *f*_01_ = 0.6 MHz, *f*_02_ = 0.3 MHz, *f* = 0.3 MHz; (**d**) *f*_01_ = 0.6 MHz, *f*_02_ = 0.3 MHz, *f* = 0.6 MHz.

In the above simulations, each composite can significantly attenuate the incident stress wave of a specified frequency. In the following simulation results, by adjusting the Young’s modulus of the coating, the particles in the red and blue rectangular regions, as shown in Figure 10, have different natural frequencies. The kinetic energies and attenuation coefficients are shown in Figure 13 and Figure 14, respectively. In the figures, Particle 1 represents the particles on the left-hand column, while Particle 2 represents the particles on the right-hand column. The natural frequencies of the two types of particles, i.e., *f*_01_ and *f*_02_, are 0.3 MHz and 0.6 MHz, respectively, for the results shown in Figure 13a,b, and 0.6 MHz and 0.3 MHz, respectively, for the results shown in Figure 13c,d. Incident stress waves with the frequencies of 0.3 MHz and 0.6 MHz are applied separately on the left-hand side boundary of the model.

As shown in Figure 13a, the kinetic energy of the particles with *f*_01_ = 0.3 MHz is significantly higher than those of the matrix and the particles with *f*_02_ = 0.6 MHz, because the frequency of the incident wave is the same as the natural frequency of the particles on the left-hand side, i.e., *f* = *f*_01_ = 0.3 MHz, and the local resonance occurs to the particles with *f*_01_ = 0.3 MHz. If the incident wave has the same frequency as the particles on the right-hand side, i.e., *f* = *f*_02_ = 0.6 MHz, as shown in Figure 13b, the kinetic energy of the particles with *f*_02_ = 0.6 MHz is significantly higher than that of the particles with *f*_01_ = 0.3 MHz, due to the local resonance of the particles with *f*_02_ = 0.6 MHz. As can be seen from the attenuation coefficients for Cases (a) and (b) in Figure 14, the composite embedded with the particles of two different natural frequencies can attenuate both the stress waves with the frequencies of 0.3 MHz and 0.6 MHz.

By switching the natural frequencies for the two types of particles in the red and blue boxes (Figure 10), i.e., *f*_01_ = 0.6 MHz and *f*_02_ = 0.3 MHz, similar attenuation behaviors are observed: larger kinetic energies are obtained for the particles with the natural frequency equal to the frequency of incident stress wave, as shown in Figure 13c,d. The attenuation coefficients for Cases (c) and (d) provided in Figure 14 also confirm this attenuation effect.

**Figure 15:**
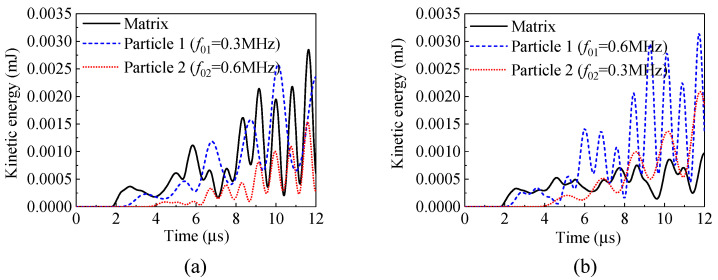
Kinetic energy (mJ): (**a**) *f*_01_ = 0.3 MHz, *f*_02_ = 0.6 MHz; (**b**) *f*_01_ = 0.6 MHz, *f*_02_ = 0.3 MHz.

In the above study, structures containing particles with two different natural frequencies have attenuation effect on both incident waves when each wave is separately loaded. However, most of the excitations are broad-banded in practice. Thus, in the study, incident stress waves with two frequencies (*f* = 0.3 MHz, *f* = 0.6 MHz) are introduced at the same time, and the model is the same as that in Figure 10. As can be seen from the kinetic energies of the matrix and particles in Figure 15, in the two different samples, the kinetic energies of the particles are both significant, indicating that the local resonance of particles are inspired.

**Figure 16:**
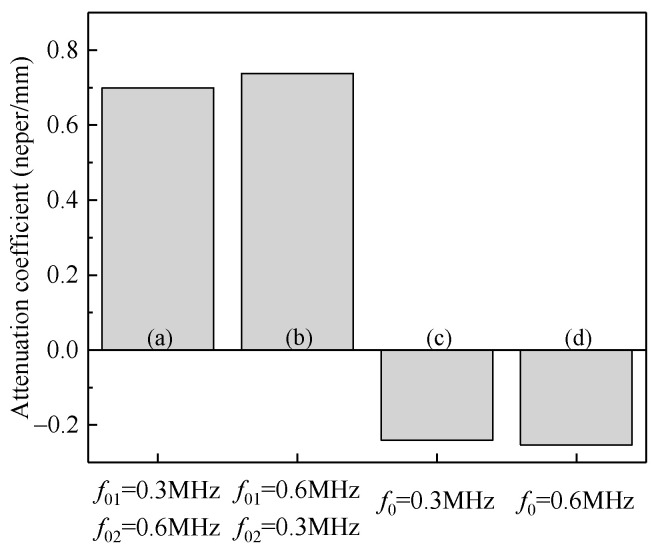
Attenuation coefficients of the composites under broad-band stress waves: (**a**) *f*_01_ = 0.3 MHz, *f*_02_ = 0.6 MHz; (**b**) *f*_01_ = 0.6 MHz, *f*_02_ = 0.3 MHz; (**c**) *f*_0_ = 0.3 MHz; (**d**) *f*_0_ = 0.6 MHz.

In terms of the attenuation coefficient as shown in Figure 16, the attenuation effect on the stress waves through local resonance is also obtained. It can be seen that different particle arrangements have little influence on the attenuation coefficient. By contrast, if there is only one type of particles presented in the matrix, the amplitude of the stress wave is not attenuated, although local resonance can occur, because the stress wave with another frequency can still propagate through the composite. Therefore, to attenuate a wide band of stress waves, a corresponding distribution of the natural frequencies of particles is required. A variety of stress waves are attenuated by adding a variety of particles to the composite.

## 5. Conclusions

The local resonant attenuation of stress wave in particulate composites is discussed in this work. The coated particles are uniformly distributed inside the composite. The local vibration mode and the natural frequency of the particles are studied numerically. The effects of particle diameter, density, Young’s modulus and the thickness of the coating layer on the natural frequency are examined.

Previous studies focused on the stress waves with low frequencies in the order of kHz, while higher frequencies of stress waves propagating through a particulate composite are investigated in the present work. The mechanism of the stress wave attenuation by adding solid particles is revealed. When the frequency of the excitation is close to the natural frequency associated with the local vibration mode, the local resonance of the particles is agitated. As a result, a large amount of the incident energy transforms to the kinetic energy of the particles, which is thereafter dissipated in the process of the strong particle oscillation.

The FEM simulations show that a particulate composite exhibits a much better attenuation performance for the stress wave, which has the frequency close to the natural frequency of the particles. It is also demonstrated that the attenuation for the incident stress waves with a broad distribution of frequencies can be achieved by introducing particles with various local natural frequencies in a composite.

## Figures and Tables

**Table 1:** Material parameters.

Material	Density (kg/m^3^)	Young’s Modulus (MPa)	Poisson Ratio
Matrix	1070	70	0.465
Particle	2500	65,000	0.25
Coating	1300	0.118	0.469

## Data Availability

The data presented in this study are available on request from the corresponding author.
